# Metabolomic Profiling and Assessment of Phenolic Compounds Derived from *Vitis davidii* Foex Cane and Stem Extracts

**DOI:** 10.3390/ijms232314873

**Published:** 2022-11-28

**Authors:** Jianhui Cheng, Jiang Xiang, Lingzhu Wei, Ting Zheng, Jiang Wu

**Affiliations:** Institute of Horticulture, Zhejiang Academy of Agricultural Sciences, Hangzhou 310021, China

**Keywords:** *V. davidii* Foex, phenolic compounds, antioxidative properties, cane and stem extracts

## Abstract

Phenolic extracts from berry seeds have been extensively studied for their health benefits. However, few studies have been conducted on the effects of phenolic extracts from *Vitis* L. canes and berry stems. The Chinese spine grape (*V. davidii* Foex) is an important and widely distributed wild species of *Vitis* L. The present study explored the metabolomic profile and evaluated the antioxidant activity of phenolic compounds in extracts from *V. davidii* Foex. canes and stems, with a focus on their role in preventing DNA damage caused by free radicals and inhibiting the growth of breast (MCF-7) and cervical (HeLa) cancer cells. Total phenolic compounds in the dried berry stems of spine grapes were higher than that in vine canes. Analysis of the extracts showed that proanthocyanins, epicatechin, catechin, and phenolic acid were the main phenolic compounds in *V. davidii* Foex, but in higher quantities in berry stems than in vine canes. However, *trans*-resveratrol and kaempferol 3-*O*-glucoside were present in the vine canes but not in the berry stems. Antioxidant analysis by FRAP and ABTS showed that extracts from berry stems and vine canes had a higher antioxidant activity than thinned young fruit shoots before flowering, leaves, peel, pulp, and seeds in *V. davidii* Foex. Moreover, the antioxidant activity of extracts from berry stems was higher than that in other grape species, except for muscadine. In vitro analyses further showed that the extracts significantly increased H_2_O_2_ scavenging ability and conferred a protective effect against DNA damage. Furthermore, a low concentration of phenolic compounds in extracts from the vine canes and berry stems of spine grapes inhibited the proliferation of the MCF-7 and Hela cancer cells. These research results provided some important useful information for the exploitation of *V. davidii* Foex canes and berry stems and indicated that canes and stems of *V. davidii* Foex had good antioxidant properties, anticancer activity and prevented DNA damage, providing evidence for medical utilization of *V. davidii* Foex.

## 1. Introduction

Grapes (*Vitis* L.) are woody vines known for their rich flavor and highly nutritious properties, hence playing an important role in the production of fruit trees across the world. Expectedly, grape extracts have gained popularity over the recent years in both medicine and health. The beneficial effects of grapes and related products are attributed to the biological compounds that they contain. Among these compounds, there are phenolic compounds such as phenolic acids, anthocyanins, catechins, resveratrol, and procyanidins. According to recent reports, phenolic compounds in grapes are associated with anti-aging [1], safeguarding against reactive oxygen species-induced DNA damage [2], anti-inflammatory effects [3,4], anticarcinogenic activity [5,6], antifungal activity [7], and neuroprotection [8], as well as promoting gut health [9]. However, most research on grape extracts has been carried out on seeds, peels, and pomace [10,11,12]. Dormant vines are pruned every year, and the pruned canes are discarded on the ground to rot, smashed back into the field, or burned in China’s grape regions. During vinification, grape stems produce large quantities of byproducts that have been little studied and less valued. However, this byproduct has been demonstrated to contain high-value bioactive compounds, including flavonoids, stilbenes, and phenolic acids [13,14,15]. Their exploitation and utilization as a raw material is therefore an interesting gap in research that needs to be explored.

Spine grapes (*V. davidii* Foex), a species of vine native to East Asia, thrives in high temperature and high humidity environments, is disease resistant, has low light resistance and accounts for part of the area used for grape cultivation in China [16]. Analysis of the scavenging capacity of wines made from spine grapes has revealed their strong antioxidant properties [17]. A few studies have investigated the biological activity of *Vitis* L. stem or cane extracts [2,18,19], but those especially from *V. davidii* Foex have not been extensively investigated and the differences among *Vitis* L. species is not well understood. Moreover, phenolic compounds play an important role in resisting adverse conditions in vivo and in vitro of *Vitis* L. and their synthesis has been shown to be affected by environmental and climatic conditions [20,21]. Therefore, the objective of the present study was to evaluate the phenolic content and antioxidative properties of vine cane and stem extracts from *V. davidii* Foex grown under humid subtropical climatic conditions, particularly in the prevention of DNA damage caused by free radicals and the inhibition of breast (MCF-7) and cervical (HeLa) cancer cell growth. In addition, we also compared the phenolic content and antioxidative properties of cane and stem extracts between *V. davidii* Foex and other species.

## 2. Results and Discussion

### 2.1. Metabolic Profiles of Vine Canes and Berry Stem Extracts

**Figure 1:**
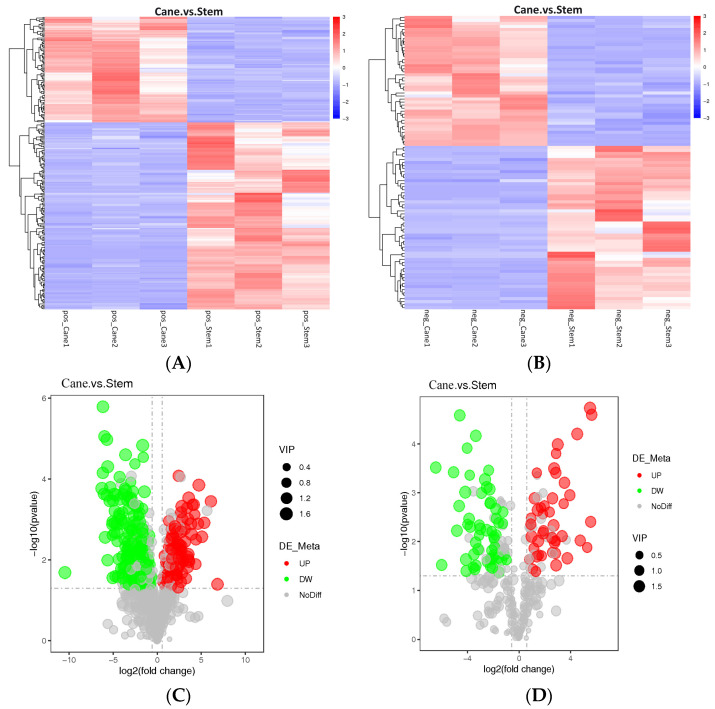
An overview of the metabolic profiles of *V. davidii* Foex cane and stem samples. (**A**) Cluster heatmaps of different samples, generated based on various metabolites, in the positive mode. (**B**) Cluster heatmaps of different samples, generated based on various metabolites, in the negative mode. (**C**,**D**) Comparative metabolic profile volcano diagram. The red, green, and gray circles represent the up-regulated, down-regulated, and insignificant metabolites, respectively. The horizontal axis represents the fold change in metabolites, and the vertical axis represents the significance level. Comparison of Sample1. vs. Sample2 illustrates the differences between metabolites in the former (Sample1) and the latter (Sample2).

Among the 975 metabolites found in this study, 689 were in the positive mode whereas 286 were in the negative mode (Table S1). The various metabolites are presented as cluster heat maps, shown in Figure 1A,B. In addition, differential metabolites were detected in both vine canes and grape stems (Figure 1C,D). Compared to the grape stems, positive and negative modes of analyses showed a decrease in the content of 154 and 54 metabolites, respectively, in vine canes. However, there was an increase in the content of 87 and 43 metabolites, respectively, in vine canes.

**Figure 2:**
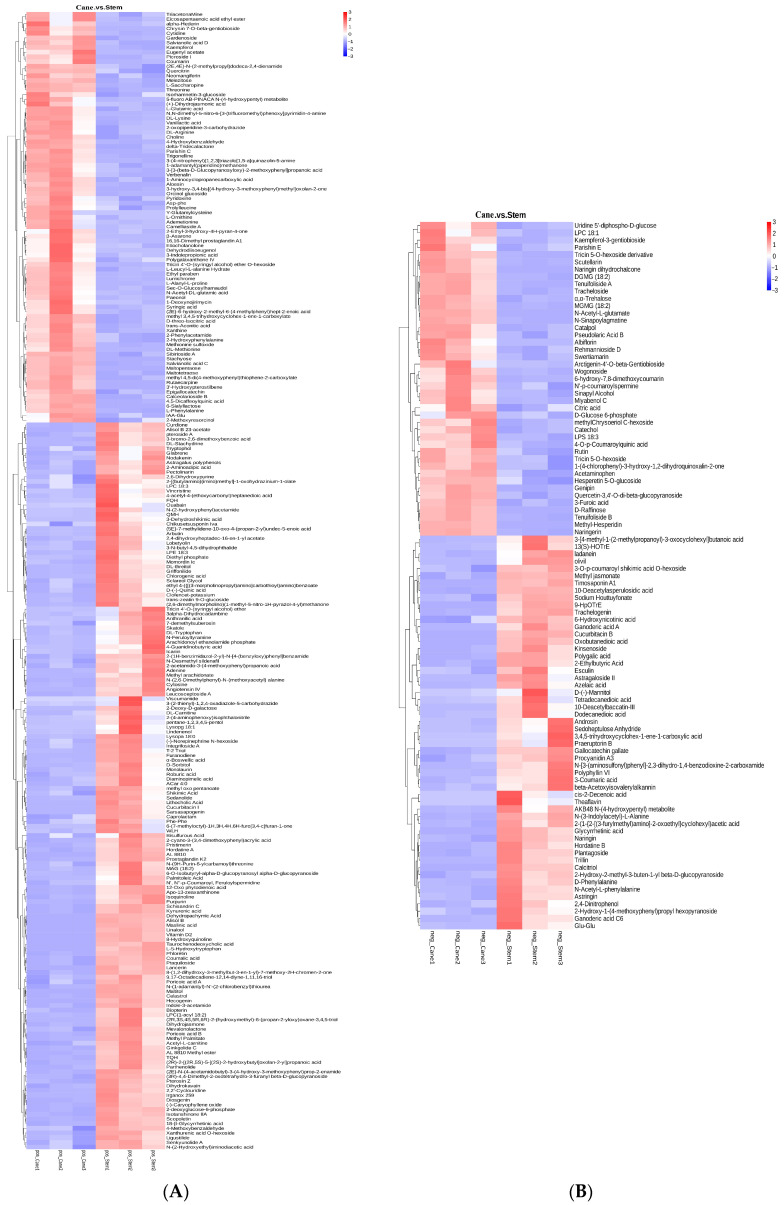
Comparison of differential metabolites in *V. davidii* Foex canes and stems. (**A**,**B**) Differential metabolites in the positive and negative modes, respectively.

The main nutrients in grapes are phenolic compounds, which primarily contain flavonoids and nonflavonoids, including flavonols, flavanols, anthocyanins, stilbenes, hydroxycinnamic acids, and hydroxybenzoic acids [22,23]. An analysis of the metabolomic profile revealed the presence of 118 compounds in extracts from *V. amurensis* berries [24]. In addition, it was reported that anthocyanin levels increased during berry maturation and ripening whereas polymeric compounds decreased [25]. The six flavanols, seven flavonols and three phenolic acids were found in pruned canes of from wild *Vitis* accessions and *Vitis vinifera* cultivars [26]. In our study, the differential metabolites obtained in vine canes and berry stems are listed in Figure 2. In the canes, there was an increase in the content of various flavonoids, including kaempferol, camelliaside A, epigallocatechin, quercitrin, naringin dihydrochalcone, naringerin, rutin, quercetin-3,4′-*O*-di-beta-glucopyranoside, hesperetin 5-*O*-glucoside. However, the levels of other flavonoids, including phloretin, purpurin, glabrene, hecogenin, ladanein, procyanidin A3, naringin, 3-coumaric acid, gallocatechin gallate, plantagoside, theaflavin, decreased. These flavonoids contribute significantly to the antioxidant activity of cane and stem extracts.

### 2.2. Composition of Phenolic Compounds

**Figure 3:**
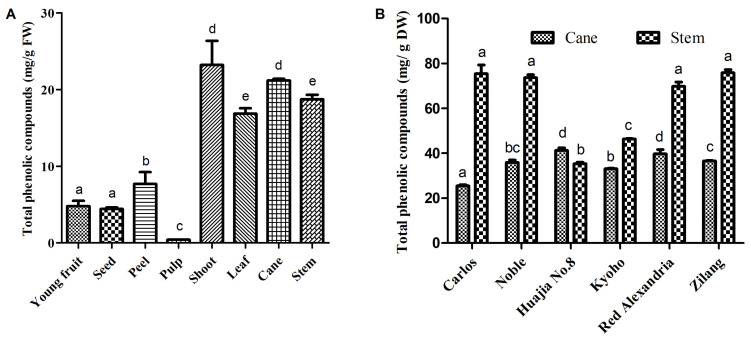
Total phenolic content in the different fresh tissues of *V.davidii* Foex (**A**), and the dried cane and stem extracts from different *Vitis* L. varieties (**B**). Bars with different letters are significantly different (*p* < 0.05).

The total phenolic compounds (TPC) in the different tissues of *V. davidii* Foex varied significantly. Higher levels of total phenolic compounds were mainly observed in the vine canes and berry stems of *V. davidii* Foex cv Zilang, whereas very low concentrations were isolated from young fruits, peel, seeds, and pulp (Figure 3A). The total phenolic compounds in fresh tissues occurred in the following decreasing order: shoot, cane, stem, leaf, berry peel, young fruit, seed, and berry pulp. In addition, the total phenolic compounds in dried canes and stems varied significantly among the grape cultivars studied (Figure 3B). Notably, *V. davidii* Foex cv Zilang and Muscadine cv Carlos and Noble had the highest levels of total phenolic compounds in grape stems (75.8 mg/g DW, 75.4 mg/g DW, 73.6 mg/g DW respectively), followed by *V. vinifera* cv Red Alexandria (69.8 mg/g DW), while the lowest quantities were observed in *V. pseudoreticulata* L.-*V. vinifera* L. cv Huajia No. 8 (35.3 mg/g DW). In vine canes, the TPC of *V. davidii* Foex cv Zilang corresponded with the middle value (36.6 mg/g DW). Pantelić et al. [12] reported that the content of phenolic compounds in berries was in the order of seed, peel, and pulp in 13 grapevine varieties grown in Serbia. It is also documented that berry seeds have the highest content of flavan-3-ols, particularly gallocatechin gallate and catechin gallate. However, flavonols and anthocyanins are more abundant in skins. In this study, the content of phenolic compounds in the seeds of *V. davidii* Foex cv Zilang was lower than that in the peels. According to a previous report, the seeds of *V. palmata*, *V. vinifera*, and *V. vulpina* had significantly higher levels of total polyphenols than other species [27]. Similarly, existing literature shows that the TPC of vine canes and berry stems varies greatly among species. It was also reported that the levels of polyphenols in the ripe berries of 147 grape accessions from 16 wild *Vitis* L. species were about 2 to 10 folds higher than those in *V. vinifera* [28]. Furthermore, previous research found that the extracts of muscadines had high levels of total phenolic content and possessed strong antioxidant activities [29]. In this study, the content of phenolic substances in the berry stems of *V. davidii* Foex was comparable to that of muscadines, suggesting that it can be used as a better raw material for the extraction of polyphenols.

High performance liquid chromatography (HPLC) analysis showed that different classes of polyphenols such as flavonoids and phenolic acids were present in the vine canes and berry stems of *V. davidii* Foex (Table 1). Notably, the main phenolic substances in vines canes were proanthocyanins (3.25 mg/g DW), epicatechin (3.15 mg/g DW) and catechin (1.05 mg/g DW). Proanthocyanins, epicatechin, and catechin were also the main phenolic substances in stems, which occurred at higher levels (11.29 mg/g DW, 10.43 mg/g DW, and 2.16 mg/g DW, respectively). Numerous studies have shown that grapes are rich in polyphenolic bioactive compounds. For instance, a previous assessment of polyphenols in 344 European grape (*V. vinifera*) cultivars through HPLC-MS found 36 polyphenolic compounds, including 16 anthocyanins, 6 flavonols, 6 flavanols, 6 hydroxycinnamic acids, and 2 hydroxybenzoic acids. In addition to anthocyanins, the content of flavanols in the berries was higher [28]. It was also reported that flavan-3-ols were the most abundant polyphenols in seeds, and collectively accounted for more than 96% of the total phenolic content [30]. Moreover, a recent study showed that gallic acid, proanthocyanidins, and ellagic acid were the main phenolics in muscadines [29]. In this study, flavanols including proanthocyanins and epicatechin were also abundant in vine canes and berry stems.

### 2.3. Antioxidant Activity

**Figure 4:**
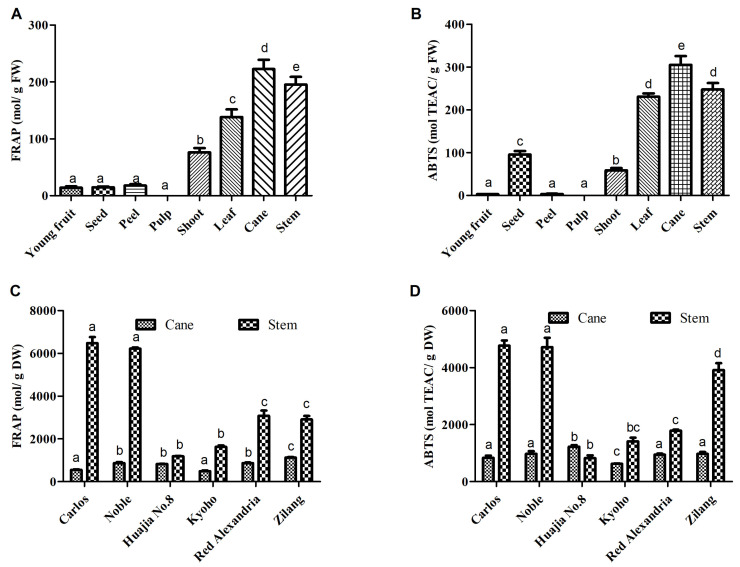
Total antioxidant activities of extracts from different fresh tissues of *V. davidii* Foex (**A**,**B**) and from the dried cane and stem extracts of different *Vitis* L. varieties (**C**,**D**) determined by FRAP and ABTS. Bars with different letters are significantly different (*p* < 0.05).

In the present study, FRAP (ferric ion reducing antioxidant power) and ABTS [2,2′-azinobis-(3-ethylbenzothiazoline-6-sulfonic acid)] assays were used to determine the antioxidant activities of extracts from the canes and stems of *V. davidii* Foex cv Zilang. The assays revealed a significant difference in the antioxidant capacities of different tissues from *V. davidii* Foex. Although the pulp is the edible part of grapes, it was associated with the lowest antioxidant activity. However, phenolic compounds in extracts from canes and stems had the strongest antioxidant activities, compared with young fruits, peels, pulp and the seeds of mature fruits (Figure 4A,B). The study also found significant differences in antioxidant capacity among grape varieties. For example, the antioxidant capacity of the stem of Muscadine cv Carlos and Noble was higher than that of other varieties (Figure 4C,D), followed by *V. vinifera* cv Red Alexandria and *V. davidii* Foex cv Zilang, respectively. Similar results were obtained when the ABTS assay was used to assess antioxidant activity. These findings were similar to those obtained by Apostolou et al. and Zhang et al. [2,19] who reported that extracts from grape canes contain a considerable amount of phenolic compounds and possess strong antioxidant activities in vitro. The results from this study, therefore, suggest that extracts from berry stems and vine canes may be useful as natural antioxidants in food and pharmaceutical products.

### 2.4. DNA Damage-Preventing Activity against Reactive Oxygen Species (ROS)

**Figure 5:**
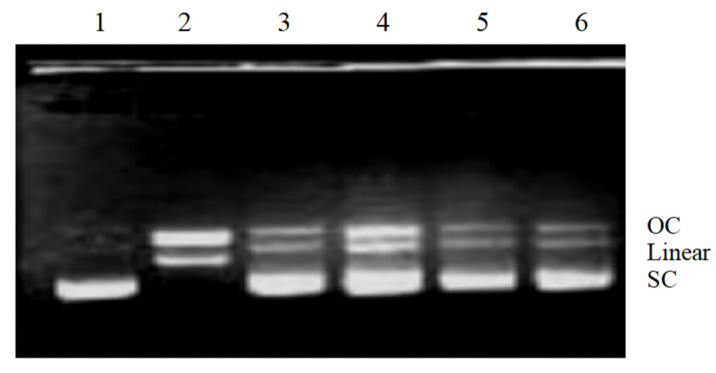
Electrophoretic analysis of DNA breaks generated by the Fenton reaction on agarose gels. Lane 1, pBR322 DNA; Lane 2, DNA damage control (DNA treated with FeSO_4_ and H_2_O_2_); lanes 3–4 and 5–6, DNA treated with FeSO_4_ and H_2_O_2_ in the presence of phenolic compounds (0.15 mg/mL and 0.3 mg/mL) from *V. davidii* Foex canes and stems, respectively.

The effects of phenolic compounds extracted from *V. davidii* grape canes and stems on DNA were also investigated in vitro. Notably, the Fenton reaction produces hydroxyl radicals, which break supercoiled plasmid DNA into three forms: supercoiled (SC), open circular (OC), and linear (Linear). Figure 5 shows the effect of phenolic compounds on OH-induced DNA damage. In the DNA control (Lane 1), the SC form was broken down into the OC and Linear forms by the Fenton reaction (Lane 2). A higher recovery of the SC form (Lane 3–6) suggested that phenolic compounds protected DNA from damage, contrary to the extent of DNA damage observed in Lane 2 of Figure 5. Due to their stronger ROS-scavenging activity, phenolic compounds in *V. davidii* Foex canes and stems protect DNA in vitro. Moreover, existing evidence shows that DNA damage is extremely disruptive to the transmission of genetic information, either by causing cell death or triggering mutations. Therefore, if not repaired on time, DNA damage poses a serious threat to cells and may result in tumors, immunodeficiency, and diseases in multicellular organisms [31]. In this study, it was shown that grape stem extracts were protective against hydroxyl radical-induced DNA damage, corroborating the findings from a previous study [2]. As such, it is important to scavenge H_2_O_2_ and protect biological systems by consuming natural antioxidants.

### 2.5. Inhibition of Cancer Cell Growth in the MCF-7 and HeLa Lines

**Figure 6:**
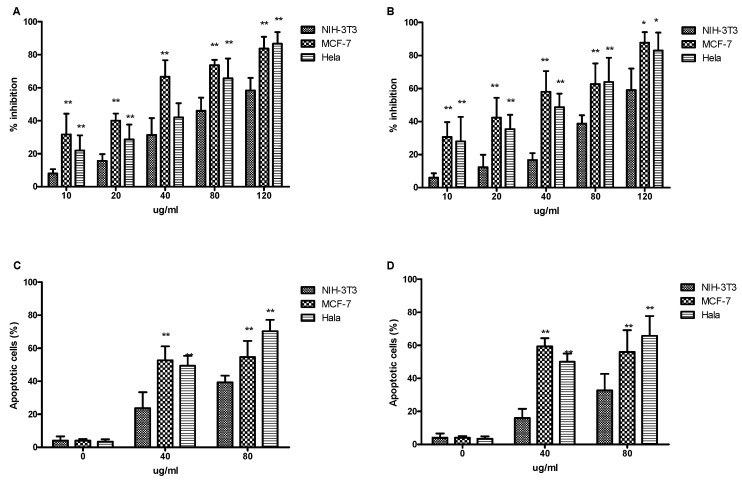
The effects of phenolic compound extracts from *V. davidii* Foex canes and stems on the growth and apoptosis of cancer cells. (**A**,**C**) canes; (**B**,**D**) stems. * Indicates significant difference (*p* < 0.05); ** Indicates extremely significant difference (*p* < 0.01).

Phosphatidylserine translocates to the exterior surfaces of the plasma membrane after binding to annexin V-FITC, indicating early apoptosis. Damaged or fragmented DNA is permeable to PI after cell death occurs. Additionally, cells stained with annexin V and PI only enter the plasma membrane when it has deteriorated. Results from the present study showed that the phenolic compounds in extracts from vine canes and grape stems had significant inhibitory effects on the proliferation of MCF-7 and Hela cells, in a dose-dependent manner (10–120 µg/mL), compared with the normal NIH-3T3 cells (Figure 6A,B). Moreover, both the cane and stem extracts promoted the apoptosis of MCF-7 and Hela cancer cells. At a concentration of 40 µg/mL, the extracts had a good effect on MCF-7 cells and had minimal side effect on the normal NIH-3T3 cells. The effect on Hela cells increased with concentration, and the higher the effect, the greater the difference was, compared with the normal NIH-3T3 cells (Figure 6C,D). In this assay, the rate of apoptotic cell death following drug exposure was quantitatively estimated. Grape antioxidants have received increasing attention for their potential anticancer effects, and several studies have shown that they are linked to a reduced risk of cancer, including breast and colon cancer [32,33]. Furthermore, it was previously shown that extracts from grape stems and berries inhibited the growth of liver (HepG2) cancer cells [2,34]. Our results showed that the lower concentrations of phenolic compounds in extracts from vine canes and grape stems inhibited the growth of MCF-7 and HeLa cells.

## 3. Materials and Methods

### 3.1. Characteristics of the Experimental Site

The study was conducted in the vineyard of Zhejiang Academy of Agricultural Science, China (120°24′ E, 30°26′ N). The soils in this area are predominantly marine-florigenic yellow loamy paddy soil, with a pH of 6.8. The average annual air temperature is 17.6 °C. The vineyard has an average rainfall of 1400 mm per year, with about 40% of this rainfall received in June, July, and August.

### 3.2. Materials

The samples studied were thinned young fruits, pruned shoots, leaves, peel, pulp, seeds, vine canes and berry stems obtained from *V. davidii* Foex (Zilang) in 2019, canes and stems from *V. rotundifolia* Michx. (Carlos and Noble), *V. vinifera* (Red Alexandria), *V. pseudoreticulata-V. vinifera* (Huajia No. 8) and *V. vinifera-V. labrusca* (Kyoho). Shoots from *V. davidii* Foex were directly obtained during the manual topping processes before flowering. Young fruits were procured by fruit thinning at 20 days after flowering, and these immature fruits usually were discarded. The leaves and berries of *V. davidii* Foex were collected at the maturity stage. Peel, pulp, seeds, and stems were obtained by manual separation and stored at −70 °C until use. Canes were collected during pruning in winter. In addition, vine canes and de-separated berry stems were air-dried and ground into powder and stored at room temperature (20–25 °C).

### 3.3. Metabolomic Profiling and Analysis

The vine canes and berry stems samples (three replicates, 100 mg per replicate) were individually grounded with liquid nitrogen and the homogenate was resuspended with 500 µL prechilled 80% methanol by well vortex, and then incubated on ice for 5 min and centrifugated at 15,000× *g*, 4 °C for 20 min. The supernatant was diluted to final concentration containing 53% methanol by LC-MS grade water and analyzed through UHPLC-ESI-MS, which was conducted using a Vanquish UHPLC system coupled with an Orbitrap Q ExactiveTM HF-X mass spectrometer (Thermo Fisher, Dreieich, Germany). Metabolic components were separated using a Hypersil Gold column (100 mm × 2.1 mm, 1.9 µm particle size). In the positive polarity mode, elusion was performed with eluents A (0.1% formic acid) and B (methanol), whereas in the negative polarity mode, elusion was conducted with eluents A (5 mM ammonium acetate, pH 9.0) and B (Methanol). The following conditions were used for the solvent gradient: 0–1.5 min, 2% B; 1.5–3 min, 2–85% B; 3–10 min, 100% B; 10–10.1 min, 100–2% B; 10.1–12 min, 2% B; followed by 5 min of re-equilibration. In the positive and negative polarity modes, the Q ExactiveTM HF mass spectrometer was operated at 3.5 kV in a spray voltage, 320 °C in capillary temperature, 35 psi in sheath gas flow, 10 L/min aux gas flow rate, and 60 RF levels at the S-lens and 350 °C Aux gas heater temperature.

Peak picking, peak alignment, and quantitation of each metabolite in the raw data files generated by UHPLC-MS/MS were conducted using ThermoFisher’s Compound Discoverer 3.1. Quality assessment of the metabolites was accomplished by comparing their retention time, m/z, and ion peak mode with those obtained from standard databases, the mzCloud database https://www.mzcloud.org/ (accessed on 20 July 2020), and the Mass Bank database https://www.massbank.jp/ (accessed on 20 July 2020). The peak area was used for quantitative analysis. Additionally, QC samples were used to evaluate the stability of the system, and blank samples were utilized to remove background ions. Statistical analysis was performed using R (version 3.4.3), Python (version 2.7.6), and CentOS (release 6.6). Variable Importance in the Projection (VIP), fold change (FC), and *p*-values are used to identify the differentially accumulated metabolites between different samples. Notably, the PLS-DA model uses the VIP to represent the variable projection importance of the first principal component, which represents the contribution of metabolites to group classification [35]. In the comparison group, FC represented the ratio between the quantitative means of each metabolite. Univariate analysis (*t*-test) was used to calculate the *p* values by Multi Experiment Viewer 4.0. The metabolites were considered differential whenever VIP > 1, *p*-value = 0.05, fold change = 2 or FC = 0.5. Moreover, a volcano plot was created using ggplot2 in R to filter metabolites of interest based on log2 (fold change) and -log10 (*p*-value). The Kyoto Encyclopedia of Genes and Genomes (KEGG) was also used to first annotate differential metabolites (Figure S1).

### 3.4. Extraction of Phenolic Compounds

As previously described [36], total phenolic compounds were extracted from all kinds of fresh tissues and dried samples in five replicates. The extracts were then stored at 20 °C in the dark, before being analyzed further for phenolic content, as previously described [37,38]. According to the calibration curve, the content was expressed as milligrams of gallic acid equivalents per gram of tissue.

### 3.5. Analysis of Polyphenols by HPLC

Phenolic compounds were qualitatively and quantitatively analyzed, according to a previous report [28]. The HPLC analysis was performed using an autosampler, binary pump, column compartment, diode array detector and the Waters BreezeTM software (Waters HPLC 1525, Milford, CT, USA). Additionally, separation of phenolic compounds was carried out on a Waters SB-C18 column (150 mm × 4.6 mm, 5 µm particle size). Briefly, 1 mL extract was filtered through 0.45 µm filter units and 15 µL was injected directly without purification. Mobile phase A (aqueous solution of 10% formic acid) and mobile phase B (aqueous solution of acetonitrile/formic acid, 90:10, *v*/*v*) were used for elution. The flow rate was maintained at 1 mL/min with a linear gradient as follows: 0–25 min, 5–15% B; 26–53 min, 15–27% B; 54–57 min, 27–5% B; followed by 5 min of re-equilibration of the column before the next run. All samples were analyzed simultaneously at 280, 320, and 365 nm, and three replicate experiments were conducted for each sample. By comparing the UV spectra and retention times with the reference compounds, the peaks of the samples were identified and quantified. Methanol was used as the solvent for all standards.

### 3.6. Measurement of Antioxidant Properties by ABTS and FRAP

The ABTS scavenging capability was determined as reported by Miller et al. [39]. Briefly, the ABTS (7 mM) solution was oxidized for 16 h in the dark with potassium peroxodisulfate (2.45 mM) at room temperature. A solution of ABTS^+^ was diluted with 80% ethanol and measured at 734 nm (Molecular Devices, SpectraMax M5, San Jose, CA, USA). After extract samples (10 µL) was mixed with 200 µL of the ABTS+ solution for 5 min at 30 °C, absorbance at 734 nm was determined. Trolox was used as a reference standard. The percentage inhibition of ABTS^+^ of the test sample and known Trolox solutions was then calculated using the following formula:

Inhibition (%) = (A_0_ − A_1_) × 100/A_0_.

This sample’s initial absorbance was A_0_, and its final absorbance was A_1_. Moreover, the Trolox equivalent antioxidant capacity (mM TEAC/g) was calculated based on the percentage inhibition of each test sample. Standard solutions of Trolox were prepared at concentrations ranging from 0.15 to 1.5 mM.

The FRAP assay used by Benzie and Strain (1996) was modified to measure the reduction of ferric ions in the sample extracts [40]. Antioxidant properties were measured in mM ferrous equivalents per gram of tissue. The standard calibration curve was prepared using a ferrous sulphate solution ranging from 0.15 to 1.5 mM.

### 3.7. Hydroxyl Radical-Induced DNA Strand Cleavage Assay

A reaction was performed in a tube filled with pBR322 DNA (0.25 g) in 50 mM phosphate buffer (pH 7.4), 2 mM FeSO_4_ and the test samples, to evaluate how phenolic compounds affect DNA damage caused by hydroxyl radicals, in vitro. Thereafter, 3% H_2_O_2_ was added to the mixture and incubated at 37 °C for 30 min [41]. Reaction products were then separated through electrophoresis on 0.8% agarose gels and then which the products of electrophoresis were visualized under the UV lamp of a gel imaging system (BIO-RAD, Hercules, CA, USA). All experiments were conducted in the dark to prevent photoexcitation of samples.

### 3.8. Anticancer Activity

#### 3.8.1. Cell Culture Conditions and Reagents

The human cervical cancer (HeLa), human breast cancer (MCF-7) and mouse embryonic fibroblast (NIH3T3) cell lines were obtained from Dr. Wang (Hangzhou Normal University, China). All cells were cultured at 37 °C in 5% CO_2_, in normal Dulbecco′s modified Eagle medium (DMEM) containing 10% (*v*/*v*) fetal bovine serum, in disposable plastic tissue culture flasks.

#### 3.8.2. MTT Assay for Inhibition of Cell Proliferation

The proliferative activity of cells was assessed using the MTT [3-(4,5-dimethylthiazol-2-yl)-2,5-diphenyltetrazolium bromide] assay. Briefly, 3 types of tumor cells in the logarithmic growth phase were taken respectively, their density adjusted to 5 × 10^4^/mL, and 100 µL inoculated in each well of a 96-well plate. After 24 h of adherent growth, 1 µL of the above samples (at different concentrations) was added. The final concentration of each sample is 10, 20, 40, 80, and 120 µg/mL. Three replicate wells were set for each sample concentration. In addition, dimethyl sulfoxide (DMSO) without extract was set as the control group, and DMSO without cells was used as a blank for zero adjustment, respectively. Incubation was allowed to continue at a constant temperature for 48 h, after which 10 µL of 5 g/L MTT solution (0.5 g MTT dissolved in 100 mL of phosphate buffer solution) was added to each well. After another incubation for 4 h, the supernatant was discarded, 100 µL of DMSO solution added to each well, followed by shaking for 10 min, and finally measurement of the optical density for each well at 540 nm using a microplate reader. Data were expressed as percentages of cell inhibition using the following formula:

Inhibition rate (%) = (A_control_ − A_sample_)/A_control_ × 100.

where A_sample_ and A_control_ represented the optical density of the tested substances and the negative control, respectively.

#### 3.8.3. Assessment of Apoptotic Cells

An annexin V binding assay was performed using flow cytometry to confirm that the cells were undergoing apoptosis. Following the manufacturer’s instructions, the Annexin V-FITC/PI apoptosis detection kit (Vazyme, Nanjing, China) was used to assess for apoptosis in cells through flow cytometry (Luminex, Austin, TX, USA). Human cervical cancer cells (HeLa), human breast cancer cells (MCF-7), and normal mouse embryonic fibroblast cells (NIH3T3) were treated with a phenolic compound extract at concentrations of 0, 40, and 80 µg/mL at 37 °C for 24 h. The cells were resuspended in 300 µL of binding buffer after centrifugation and washed with cold PBS. Finally, the cells were incubated with Annexin V-FITC and PI dye for 15 min and 5 min, respectively, after which flow cytometry was conducted.

### 3.9. Statistical Analysis

Statistical analyses were conducted using GraphPad Prism 5.0 (GraphPad Software, Inc., La Jolla, CA, USA), and data were presented as the mean ± standard deviation. Differences among samples were analyzed by a one-way ANOVA (analysis of variance) followed by the Student Newman–Keuls tests. Values with *p* < 0.05 were considered to be statistically significant.

## 4. Conclusions

This study found that extracts from vine canes and berry stems were rich in phenolic compounds which were shown to have strong antioxidant properties. The significant differences among grape species were observed, and the extracts from muscadine stems had the strongest antioxidant capacity. The phenolic compounds of canes and stem extracts from *V. davidii* Foex were mainly proanthocyanins, epicatechin, catechin, and phenolic acids. In addition, the results revealed that the extracts exerted a protective effect against free radical-induced DNA damage. They also inhibited the growth of breast (MCF-7) and cervical (HeLa) cancer cells in vitro. Therefore, use of berry stems and pruned canes from *V. davidii* Foex as sources of high-value food supplements rich in phenolic compounds may not only be a potential source of natural antioxidants for functional foods but also solve the problem of environmental pollution caused by random discarding or burning.

## Figures and Tables

**Table 1:** HPLC profiles identified phenolic compounds of canes and stem extracts from *V. davidii* Foex based on retention time.

Peak Number	RT (Min)	λmax (nm)	Identity	Content (mg/g DW)	
				Canes	Stems
1	2.89	280	Proanthocyanins	3.25 ± 0.61 d	11.29 ± 1.15 f
2	4.06	280	Gallic acid	0.28 ± 0.02 a	0.6 ± 0.04 ab
3	7.01	320	Caffeic acid	0.13 ± 0.00 a	0.6 ± 0.01 ab
4	8.66	280	Catechin	1.05 ± 0.19 b	2.16 ± 0.26 c
5	10.38	331	3-coumaric acid	0.04 ± 0.00 a	0.26 ± 0.01 a
6	11.75	320	Chlorogenic acid	0.16 ± 0.00 a	0.12 ± 0.00 a
7	14.21	280	Epicatechin	3.15 ± 0.11 d	10.43 ± 1.15 e
8	27.51	365	Rutin	0.22 ± 0.01 a	0.16 ± 0.00 a
9	34.21	365	Myricetin 3-*O*-glucoside	0.33 ± 0.01 ab	0.18 ± 0.00 a
10	37.62	318	*Trans*-resveratrol	0.49 ± 0.02 ab	nt
11	55.90	360	Kaempferol 3-*O*-glucoside	0.32 ± 0.02 ab	nt

Data were analysed and different letters indicate significantly different (*p* < 0.05).

## Data Availability

The original contributions presented in the study are included in the article. Further inquiries can be directed to the corresponding author.

## Supplementary Materials

The following supporting information can be downloaded at: https://www.mdpi.com/article/10.3390/ijms232314873/s1, Figure S1: KEGG enrichment pathways of differential metabolites from *V. davidii* Foex. canes and stems in positive(A) and negative (B) ion modes; Table S1: The identified metabolites from *V. davidii* Foex. canes and stems in positive and negative ion modes.
