# Supplementary material for: Metabolomic Profiling and Assessment of Phenolic Compounds Derived from Vitis davidii Foex Cane and Stem Extracts
Source: Int J Mol Sci. 2022 Nov 28;23(23):14873. doi: 10.3390/ijms232314873 (PMC9735678; doi:10.3390/ijms232314873)
Supplement: Supplementary file 1 [file ijms-23-14873-s001.zip › Figure S1.pdf]

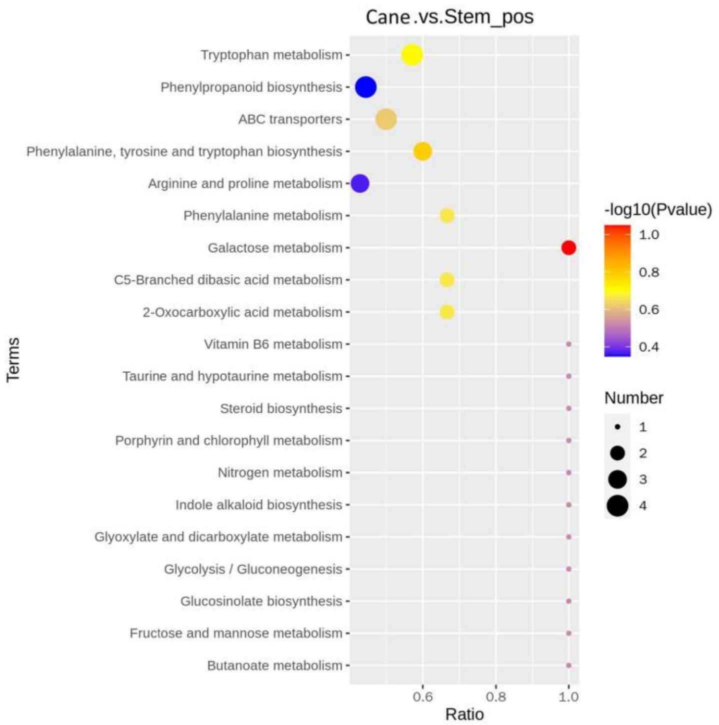

(A)

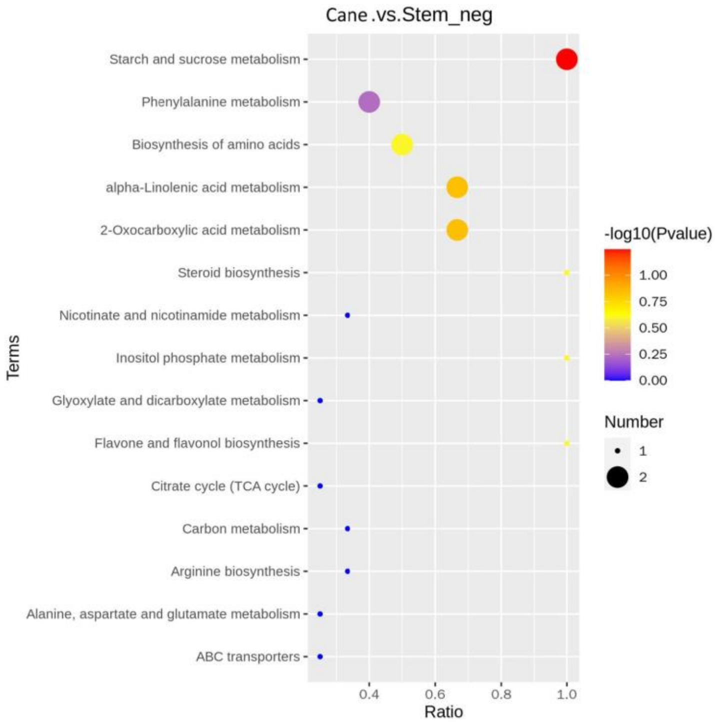

(B)

Figure S1: KEGG enrichment pathways of differential metabolites from *V. davidii* Foex. canes and stems in positive(A) and negative (B) ion modes; Table S1: The identified metabolites from *V. davidii* Foex. canes and stems in positive and negative ion modes.
